# Factors improving overall survival in breast cancer patients with leptomeningeal disease (LMD): A single institutional retrospective review

**DOI:** 10.21203/rs.3.rs-2981094/v1

**Published:** 2023-06-08

**Authors:** Gerald Wallace, Ronak Kundalia, Biwei Cao, Youngchul Kim, Inna Smalley, Peter Forsyth, Aixa Soyano, Yolanda Pina

**Affiliations:** H. Lee Moffitt Cancer Center and Research Institute; H. Lee Moffitt Cancer Center and Research Institute; H. Lee Moffitt Cancer Center and Research Institute; H. Lee Moffitt Cancer Center and Research Institute; H. Lee Moffitt Cancer Center and Research Institute; H. Lee Moffitt Cancer Center and Research Institute; H. Lee Moffitt Cancer Center and Research Institute; H. Lee Moffitt Cancer Center and Research Institute

## Abstract

**Background:**

Breast cancer-related leptomeningeal disease (BC-LMD) is a dire diagnosis for 5–8% of patients with breast cancer (BC). We conducted a retrospective review of BC-LMD patients diagnosed at Moffitt Cancer Center (MCC) from 2011–2020, to determine the changing incidence of BC-LMD, which factors impact progression of BC CNS metastasis to BC-LMD, and which factors affect OS for patients with BC-LMD

**Methods:**

Patients with BC and brain/spinal metastatic disease were identified. For those who eventually developed BC-LMD, we used Kaplan-Meier survival curve, log-rank test, univariable, and multivariate Cox proportional hazards regression model to identify factors affecting time from CNS metastasis to BC-LMD and OS.

**Results:**

128 cases of BC-LMD were identified. The proportion of BC-LMD to total BC patients was higher between 2016–2020 when compared to 2011–2015. Patients with HR + or HER2 + BC experienced longer times between CNS metastasis and LMD than patients with triple-negative breast cancer (TNBC). Systemic therapy and whole-brain radiation therapy (WBRT) prolonged progression to LMD in all patients. Hormone therapy in patients with HR + BC delayed BC-CNS metastasis to LMD progression. Lapatinib delayed progression to LMD in patients with HER2 + BC. Patients with TNBC-LMD had shorter OS compared to those with HR + and HER2 + BC-LMD. Systemic therapy, intrathecal (IT) therapy, and WBRT prolonged survival for all patients. Lapatinib and trastuzumab improved OS in patients with HER2 + BC-LMD.

**Conclusions:**

Increasing rates of BC-LMD provide treatment challenges and opportunities for clinical trials. Trials testing lapatinib and/or similar tyrosine kinase inhibitors, IT therapies, and combination treatments are urgently needed.

## Introduction

Leptomeningeal disease (LMD) is a dreadful complication occurring in approximately 5–8% of patients with breast cancer (BC)^1^. Median overall survival (OS) in untreated patients with LMD including from BC is approximately 1 month^1,2^. Aggressive treatment in breast cancer-related leptomeningeal disease (BC-LMD) can extend OS to 3–4 months, although this is BC subtype-specific^3,4^. Beyond receptor subtype stratification and examining the efficacy of HER2-targeted therapy, only a few studies have attempted to identify the clinical characteristics of BC-LMD^5–7^.

An earlier diagnosis of LMD is important to improve patient survival and enrollment in clinical trials. However, Magnetic Resonance Imaging (MRI) has a wide variability to accurately diagnose LMD,^8^ and the current gold standard of a positive CSF cytology has a very low sensitivity (< 50%) and positive predictive value.^9^ To optimize the diagnostic yield beyond traditional CSF cytology, assays that detect circulating tumor cells and cell-free DNA have been developed over the past decade, but the clinical use of these new techniques to diagnose and treat LMD has not yet been defined.^10^

Breast cancer in the central nervous system (BC CNS) has been under-recognized, and the real epidemiological data remains under-reported, especially for those who develop BC-LMD.^11^ A concerted effort to identify and treat BC-LMD at Moffitt Cancer Center (MCC) began around 2015 and is currently ongoing. This study aims to determine if the incidence of BC-LMD at MCC is changing over time, what factors may impact progression of BC CNS metastasis to LMD, and what factors may affect the OS for patients with BC-LMD.

## Methods

This project was approved by the Scientific Review Committee and Institutional Review Board at MCC (MCC #21524). A retrospective review of medical records was conducted to identify patients diagnosed and/or treated at MCC with BC and who also had a diagnosis of CNS metastases between January 1, 2011 and December 31, 2020. Only patients with confirmed diagnosis of BC and LMD were included. LMD diagnosis was confirmed with CSF cytology and/or MRI. Patients were excluded if the LMD diagnosis was based solely on clinical suspicion, if confirmatory MRI or CSF cytology was not available in the medical record, or if there was another malignancy which might seed the leptomeningeal space.

### DataCollection

Demographics, BC receptor subtype, dates of BC/CNS metastasis/LMD diagnoses, and dates of censorship/death were collected. Method of diagnosis, treatments prior to and following CNS metastasis, and treatments following BC-LMD were also collected.

### Statistical Analysis

Descriptive statistics including frequency, percentage, median, and range were calculated for patients’ demographics and clinical characteristics. Differences in continuous variables between patient groups were statistically tested using the Kruskal-Wallis tests. The associations between categorical variables and endpoints were evaluated using Chi-square test or Fisher’s exact test.

Median time between CNS metastatic disease and LMD, and OS post-LMD were estimated using the Kaplan-Meier method. Univariable Cox proportional hazards regression analysis was used to estimate hazard ratios and their 95% confidence interval (CI). Significant variables at univariable analysis were subsequently tested in multivariable Cox regression analysis. All reported *p* values were two—sided, and significance level was 0.05 (p < 0.05). Analyses were performed using R version 4.1.0.

## Results

### Cases and Demographics

**Figure 1:**
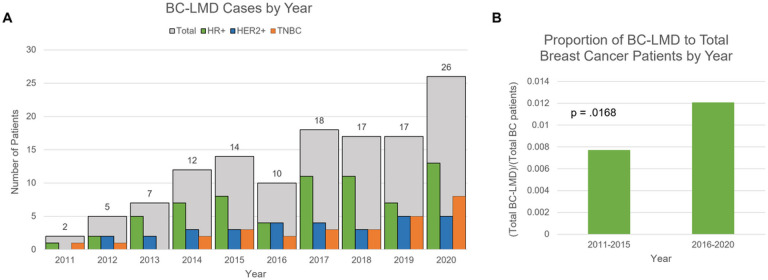
**A)** Total number of BC-LMD cases seen at Moffitt Cancer Center between 2011–2020, by breast cancer subtype. **B)** The proportion of BC-LMD patients to total breast cancer patients seen at Moffitt Cancer Center between 2011–2015 and 2016–2020. A significantly higher proportion of (BC-LMD patients)/(Total Breast Cancer Patients) was observed in the latter half of the decade (p=0.0168). **Abbreviations**: Hormone Receptor Positive (HR+); Human Epidermal Growth Factor Receptor 2-Positive (HER2+); Triple Negative Breast Cancer (TNBC); Breast Cancer Leptomeningeal Disease (BC-LMD).

One-hundred-twenty-eight patients were identified who met radiographic and/or CSF cytology criteria for BC-LMD diagnosis. Forty patients were identified between 2011–2015, whereas 88 patients were identified between 2016–2020 (Fig. 1A). The proportion of BC-LMD patients to total BC patients was significantly higher between 2016–2020 when compared to 2011–2015 (p = 0.0168, Fig. 1B). The median age of BC diagnosis was 51 years [22–79 years]. The median age of CNS metastasis diagnosis was 53 years [26–81 years] and median age of LMD diagnosis was 54 years [27–83 years].

Of the 128 patients with BC-LMD, 66 had HR + BC (52%), 34 had HER2 + BC (26%), and 28 had TNBC BC (22%). Median age at BC diagnosis significantly differed between all three groups (HR+: 55 years, [25–76]; HER2+: 50.5 years, [22–79]; TNBC: 45.5 years, [24–69]; p = 0.0348). The median age at LMD diagnosis between disease subtypes significantly differed (HR+: 58.5 years, [27–79]; HER2+: 53.5 years, [29–83]; TNBC: 48 years, [27–70]; p = 0.0023).

Out of all the patients identified, 100 (78%) had systemic metastasis prior to the diagnosis of LMD, and 114 (89%) had CNS metastasis prior to the diagnosis of LMD. Patients with HR + and HER2 + BC were more likely to develop systemic metastasis prior to the diagnosis of LMD when compared to patients with TNBC (i.e., 86% and 79%, respectively, versus 57%; p = 0.007). All three patient cohorts were equally likely to develop CNS metastatic disease prior to the diagnosis of LMD. Patients’ demographic data is summarized in Table 1.

### Factors Affecting Time between CNS Metastasis Diagnosis and LMD Diagnosis

Only patients who had at least a one-month gap between CNS metastasis and LMD diagnoses were included in the analysis. Of the 48 BC-LMD patients that met this criterion, 19 had HR + BC (40%), 19 HER2 + BC (40%), and 10 TNBC (20%). Demographics for these patients is summarized in **Table 2**, and post-CNS metastasis/pre-LMD treatment data is summarized in **Supplementary Table 1**.

**Figure 2:**
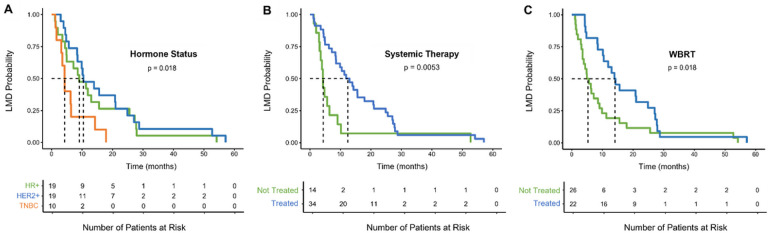
Kaplan-Meier estimates for factors affecting time between CNS-metastasis diagnosis and BC-LMD diagnosis. **A)** Patients with TNBC experienced a significantly shorter median time between breast cancer CNS-metastasis diagnosis and BC-LMD (4.3 months) compared to HR+ (9.1 months) and HER2+ (10.5 months) patients. **B)** Patients that received systemic therapy experienced a longer median time between CNS metastasis and BC-LMD (12.5 months) than patients who did not receive any systemic therapy (4.3 months). **C)** Patients that received WBRT had a longer median time between their CNS metastasis and BC-LMD diagnosis (14.1 months) compared to patients that did not receive WBRT (5.3 months). **Abbreviations**: Hormone Receptor Positive (HR+); Human Epidermal Growth Factor Receptor 2-Positive (HER2+); Triple Negative Breast Cancer (TNBC); Leptomeningeal Disease (LMD); Whole Brain Radiation Therapy (WBRT).

Patients with HR + BC and HER2 + BC experienced longer time between BC-CNS metastasis to BC-LMD diagnosis compared to patients with TNBC disease (Fig. 2A, p = 0.018). Patients who received systemic treatments post-CNS metastasis experienced a longer time to LMD diagnosis (12.5 months [8.6, 20.8)] compared to patients who did not receive systemic therapy (4.3 months [3.6, 10.2]; Fig. 2B, p = 0.0053). Furthermore, patients who received WBRT post-CNS metastasis diagnosis had a median time to LMD diagnosis of 14.1 months [10.5, 27.1], while patients who did not receive WBRT had a median time of only 5.3 months [3.6, 9.9] between their CNS metastasis and LMD diagnoses (Fig. 2C, p = 0.018).

Patients with HR + BC who received any hormone therapy had significantly longer times between their CNS metastasis diagnosis and LMD diagnosis (**Supplementary Fig. 1A**, p = 0.0005). More specifically, HR + patients that were treated with letrozole had a longer time to LMD diagnosis from CNS metastasis diagnosis (**Supplementary Fig. 1B**, p = 0.0007). Similar trends were observed in HR + BC patients who were treated with the aromatase inhibitor exemestane (**Supplementary Fig. 1C**, p = 0.0015) and CDK4/6 inhibitor palbociclib (**Supplementary Fig. 1D**, p = 0.023). Finally, the mTOR inhibitor everolimus also showed prolonged time between CNS metastasis and LMD diagnosis in HR + BC patients (**Supplementary Fig. 1E**, p = .0013). Of all these treatments, exemestane and palbociclib also showed significance in multivariate analysis (**Supplementary Fig. 1F**).

Anti-HER2 therapy lapatinib was associated with longer times between CNS metastasis and BC-LMD (**Supplementary Fig. 2**, p = 0.026) for HER2 + BC-LMD patients when compared to patients of any subtype who did not receive this treatment. Unfortunately, no systemic therapy was found to be associated with significantly longer time between CNS metastasis diagnosis to LMD diagnosis in TNBC patients.

Finally, the effects of brain tumor excision and radiosurgery were also analyzed, but patients receiving these treatments experienced no differences in the time between CNS metastasis and LMD when compared to patients who did not receive such treatments (**Supplementary Fig. 3**).

### OS with BC-LMD

Treatment data for patients post-LMD diagnosis is summarized in **Supplementary Table 2.**

**Figure 3:**
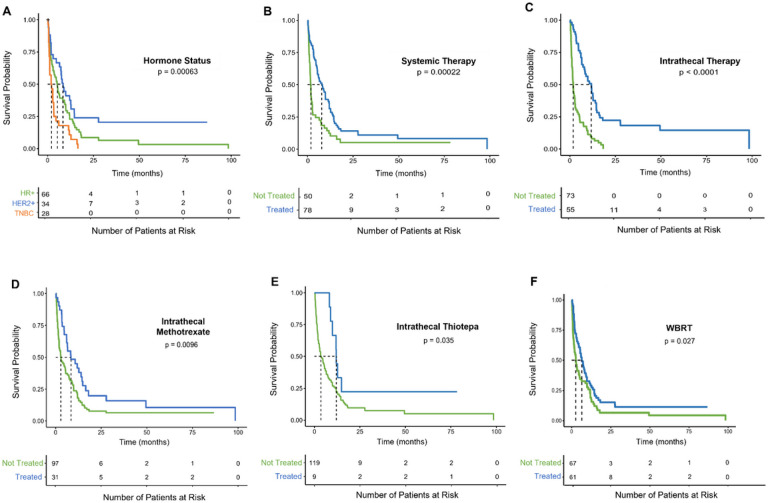
Kaplan-Meier estimate for factors affecting overall survival in BC-LMD patients. **A)** TNBC-LMD patients had a significantly lower overall survival (2 months) when compared to HR+ BC-LMD patients (5.3 months) and HER2+ BC-LMD patients (8.4 months). Median survival time between HR+ and HER2+ BC-LMD patients did not significantly differ. **B)** Patients receiving systemic therapy post BC-LMD diagnosis had a significantly higher median survival time (7.9 months) when compared to BC-LMD patients that did not receive systemic therapy (1.8 months). **C)** Patients receiving intrathecal therapy post BC-LMD diagnosis had a significantly higher median survival time (11.8 months) when compared to BC-LMD patients that did not receive intrathecal therapy (1.9 months). **D-E)** More specifically, overall survival median times were higher in patients that received intrathecal methotrexate (8.4 months) and/or intrathecal thiotepa (12 months) versus those that did not (2.9 months; 3.6 months, respectively). **F)** patients receiving WBRT post BC-LMD diagnosis had a higher overall survival median time (6.5 months) than those who did not receive WBRT (2.7 months). **Abbreviations:** Hormone Receptor Positive (HR+); Human Epidermal Growth Factor Receptor 2-Positive (HER2+); Triple Negative Breast Cancer (TNBC); Leptomeningeal Disease (LMD); Whole Brain Radiation Therapy (WBRT); Intrathecal (IT); Hazard Ratio (HR); Confidence Interval (CI).

Patients with TNBC had significantly worse median post-LMD OS (2 months, [1.1, 3.4]), when compared to both patients with HER2+ (8.4 months [6.5, 14.5]; p = 0.0016) and HR + BC (5.3 months, [2.9, 9.3]; p = .0097). Survivability by cancer subtype is summarized in Fig. 3A.

**Figure 4:**
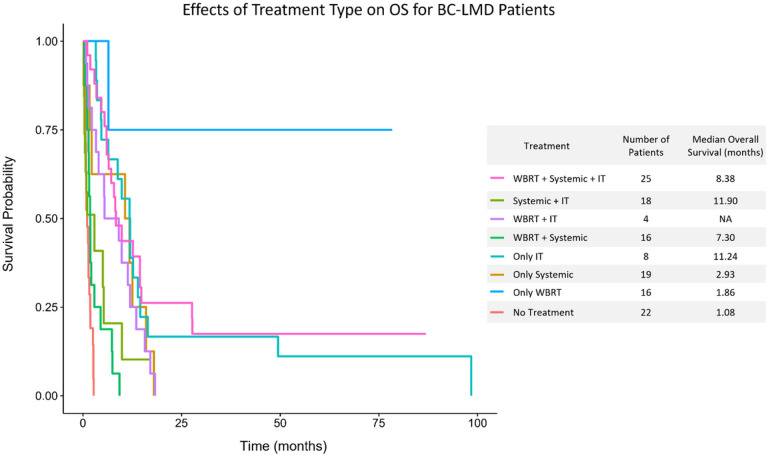
Kaplan Meier analysis of BC-LMD patients receiving different combinations of WBRT, Systemic Therapy, and/or IT therapy. Patients receiving only IT, systemic + IT, WBRT + IT, WBRT + systemic therapy, or all three therapies had a significantly longer median OS than patients that received no therapy (p<.05, respectively). Compared to WBRT alone, patients receiving WBRT + systemic therapy or WBRT + systemic therapy + IT therapy had a significantly longer median OS (p<.0001, respectively). **Abbreviations:** Breast Cancer Leptomeningeal Disease (BC-LMD); Overall Survival (OS); Intrathecal Therapy (IT); Whole Brain Radiation Therapy (WBRT).

22 patients did not receive any treatment post-LMD diagnosis, and they had a median OS of 1.08 months [0.26, 3.00]. Conversely, 106 patients received either systemic treatment, IT therapy, WBRT, or some combination of the three post BC-LMD diagnosis, and their median OS was significantly higher at 6.54 months [0.21, 106.96] (p < 0.0001). Median OS by treatment type is summarized in Fig. 4.

Fifty patients did not receive any systemic therapy post-LMD diagnosis, and they had a median OS of 1.8 months [1.4, 2.7]. Conversely, 78 patients received systemic therapy post-LMD and they had a significantly improved median OS of 7.9 months [5.5, 11.8] (Fig. 3B, p = 0.0002). However, 19 patients received only systemic therapy post LMD diagnosis. Their median OS of 2.93 month was not significantly different than patients who did not receive any treatment post-LMD (Fig. 4).

Furthermore, 55 patients received IT therapy post-LMD diagnosis, and they had a median OS of 11.8 months [8.2, 14.5]. This was significantly greater than the 1.9 month [1.4, 2.7] month median OS of patients that did not receive IT therapy (Fig. 3C, p < 0.0001). Specifically, patients receiving IT methotrexate had a 5.5 month longer median OS than patients who did not (Fig. 3D, p = 0.0096), and patients receiving IT thiotepa had a median OS that was 8.4 months longer than those who did not (Fig. 3E, p = 0.0354). Improved OS was also observed in the 8 patients who received only IT therapy for treatment post LMD diagnosis. Their median OS of 11.24 [0.21, 19.27] months was significantly greater than the OS of the 22 patients that did not receive any therapy post BC-LMD diagnosis (Fig. 4, p = 0.041).

Finally, 61 patients receiving WBRT survived a median of 6.5 [4.7, 9.8] months while 67 patients who did not receive WBRT survived a median of 2.7 months [1.7, 5.3] (Fig. 3F, p = 0.0274). However, The OS for patients that received only WBRT post-LMD diagnosis was 1.86 months [0.39, 10.11], which was not significantly greater than the OS for patients that received no therapy (Fig. 4).

In the context of HR + BC-LMD, 8 HR + patients treated with capecitabine post BC-LMD diagnosis showed median survival of 14.8 [5.5, N.E.] months while HR + patients that did not receive capecitabine showed a median survival of 4.0 [2.3, 8.2] months. However, this difference was not statistically significant (p = 0.0694). Similarly, 11 HR + BC-LMD patients treated with anthracycline showed a median OS of 12.8 (11.8, N.E.) months, which was not statistically longer than the median OS of HR + BC-LMD patients who did not receive anthracycline treatment (3.6 [2.2, 6.4] months, p = 0.0661). These marginally significant results are possibly attributable to a low power with small sample size. As noted in the collective patient survival data, IT therapy, and more specifically IT methotrexate, significantly improved OS in patients with HR + BC-LMD when compared to patients that did not receive IT therapy/IT methotrexate (**Supplementary Fig. 4A/B** p = 0.0019 and 0.0028, respectively).

For patients with HER2 + BC, systemic treatment in general, particularly anti-HER2 targeted therapies such as trastuzumab and lapatinib, significantly improved patient median survival (**Supplementary Fig. 5A-C**, p = 0.015, 0.0261 and 0.0352, respectively). HER2 + BC-LMD patients receiving IT therapy had an improved median OS by 11.1 months when compared to HER2 + BC-LMD patients that did not receive IT therapy (**Supplementary Fig. 5D**, p < 0.0001). Importantly, all HER2 + patients that received IT therapy received IT trastuzumab as a part of their treatment regimen.

Although treatment with systemic therapy collectively enhanced survival in patients with TNBC when compared to those patients who did not receive systemic therapy (3.4 months versus 1.2 months, **Supplementary Fig. 6A**, p = 0.0334), no individual therapy was found to be associated with prolonged survival in this subset of patients. However, IT therapy was found to increase OS in TNBC patients compared those that did not receive the therapy (8.9 months versus 1.1 months, **Supplementary Fig. 6B**, p = 0.001).

## Discussion

Data from MCC supports a significant rise in the number of BC-LMD cases between 2011–2020. Greater institutional efforts to identify and treat LMD patients were made in the latter half of the study period, which may have driven the increased in BC-LMD diagnosis. A slight trend towards increased proportions of patients with TNBC were seen in the current review, but all cases increased over the study period. One of the first to report this, showed that HR status influences the risk of developing LMD.^12^ This is supported by recent data suggesting HR + BC-LMD likely comprises the majority of all cases (range 48–66%), and rates of HER2 + and TNBC-LMD are more variable.^12–16^ HER2 + BC-LMD represents 14–47.4% of all cases, and TNBC-LMD varies between 13.1–40% across studies.^12–16^ The proportion of HR + patients was similar in our study at 51% compared to HER2 + BC-LMD (27%) and TNBC (22%).

BC subtype affected time between CNS metastasis and BC-LMD diagnoses. TNBC had the fastest progression to BC-LMD compared to HR + and HER2 + BC-LMD. Even though HER2 + cancer may have a predisposition for CNS invasion, a tendency to invade the leptomeninges has not been clinically revealed.^1,17^ It has been shown that increased survival in patients with BC generally correlates with increased incidence of CNS metastases.^18^ Prolonged survival and risk of developing metastases, may confer a greater risk of developing BC-LMD.^14^ Historically, HER2 + and TNBC have carried an increased risk of developing CNS metastasis, and an associated decreased survival relative to HR + disease.^19,20^ Earlier work suggested no difference among BC-LMD based on the molecular subtype.^21^ However, our findings support more recent studies demonstrating an increased risk of developing LMD for TNBC.^12,13,15,16,22^

This study also showed the use of any systemic treatment and/or WBRT post-CNS metastasis delays progression from to BC-LMD. HR + patients receiving hormone therapies and/or kinase inhibitors may experience prolonged times between CNS metastasis and LMD diagnosis. For HER2 + patients, treatment with lapatinib demonstrated similar results. However, the studies stringent criterion for stratifying patients by at least a one-month period between CNS metastasis and LMD severely reduced the total patient sample from 128 to 48 patients. Follow-up studies analyzing significantly larger patient samples is required to definitively determine factors that prolong time between CNS metastasis and BC-LMD.

Survival did not change over the study period. Five cases identified in the first two years of the study survived longer than two years, but survival over the decade remained static even accounting for these outliers. Median survival after the diagnosis of BC-LMD was 4.7 months in this study. TNBC LMD had the shortest median survival of 2 months, followed by 5.3 months in HR + and 8.4 months in HER2+. These findings concur with previous studiesand BC-LMD survival depending on BC subtype.^22,23^ While longer survival for patients with HER2 + BC-LMD likely stems from HER2 targeted systemic and IT chemotherapy.^1,17,22,24^ differences in survival in HR + or TNBC may be driven by other factors. Patients with HR + BC-LMD were five times as likely to receive systemic therapy and four times as likely to receive IT chemotherapy following BC-LMD diagnosis compared to those with TNBC. It is tempting to say, therefore, that intention to treat is the primary driver of prolonged survival, but other factors including poor performance status and extent of extracranial metastatic disease may be confounders in this data.^23^ Even so, we were unable to identify any other single factor accounting for differences in survival among patients with HR + or TNBC.

The use of systemic therapy and IT chemotherapy appeared to improve survival following the diagnosis of LMD regardless of HR subtype. IT trastuzumab showed prolonged survival in HER2 + LMD.^22,25,26^ In 2018, the first phase 1 study of IT trastuzumab showed 150 mg weekly dosing achieved steady-state levels after 1 week and was well tolerated.^27^ Prior to this publicationy, patients with HER2 + LMD treated at our institution received weekly IT doses of trastuzumab of less than 150 mg. Our study demonstrates that IT trastuzumab dosing (i.e. physiologic dosing) resulted in improved survival for patients with HER2 + disease.

To maximize IT HER2 targeted therapy, our institution opened a phase I/II study of radiotherapy followed by IT trastuzumab and pertuzumab in patients with HER2 + BC-LMD to evaluate safety and treatment outcomes (NCT04588545).^24^ Radiotherapy can eliminate tumor blockages within the leptomeninges, allowing for IT therapy to properly flow along the CSF.^28^ The current study, however, found no statistical difference in the OS between patients who received only IT therapy versus WBRT and IT therapy. However, the latter cohort included only four patients. Further investigation is warranted.

There is an increasing interest in HER2-targeting therapies in BC-LMD. Prior studies showed that pertuzumab-based therapies improved progression-free survival (PFS) when used with trastuzumab and taxanes, and when given as a first or second-line chemotherapy^29, 30^y^31^.

Of particular interest in the current study, ten HER2 + BC-LMD patients exhibited a significant increase in OS in association with systemic treatment with lapatinib, a reversible tyrosine kinase inhibitor (TKI).^32,33^ Lapatinib with capecitabine is well tolerated^34^ and improved PFS to 8.4 months versus 4.1 months using capecitabine alone.^35^ Lapatinib + capecitabine BC brain metastases showed overall response rates (ORR) of 59.1% among treatment naïve patients^36^ and 21% among patients who may or may not have been exposed to either agent previously.^37^ However, the CEREBEL trial demonstrated no difference in PFS between capecitabine plus either lapatinib or trastuzumab^38^.The EMILIA trial showed that lapatinib plus capecitabine was both less tolerable and less efficacious than trastuzumab emtansine in prolonging PFS.^39^ The LANTERN trial, a phase II trial comparing lapatinib-capecitabine versus trastuzumab-capecitabine therapy in HER2 + BC with CNS metastasis showed no significant difference in PFS but a trend favoring trastuzumab-capecitabine.^40^ Aside from two case studies, there is no prospective data describing the efficacy of lapatinib in the treatment of BC-LMD.^41,42^

Neratinib and pyrotinib are similar to lapatinib except that they irreversibly bind to the HER intracellular phosphorylase domain and have efficacy in BC brain metastases.^33^ The NALA trial compared lapatinib and neratinib and both with capecitabine in BC brain metastases and showed similar ORR (26.7% versus 32.8%, respectively), but significantly longer duration of response for neratinib rather than lapatinib (8.5 versus 5.6 months, respectively). The benefits of these TKIs were overshadowed by the combination of T-DM1 and tucatinib, as tolerability was better and efficacy was at least as good for these agents.^16, 33^ Neratinib + capecitabine enhanced OS to 10 months and improved neurological symptoms in 60% of patients with HER2 + BC LMD.^43^

Tucatinib is a newer TKIthat has shown activity in HER2 + BC brain metastases, when combined with trastuzumab and capecitabine.^44–46^ CSF pharmacokinetic analysis revealed detectable levels of tucatinib within 2 hours of administration (NCT03501979).^47^ Further studies using TKIs in BC-LMD are needed. Other studies evaluated these agents in CNS metastases and the ORR in BC-LMD was not established.

At the present time, there is a lack of effective treatments in TNBC-LMD. The current study showed an association between IT therapy and OS among BC-LMD patients, supporting prior studies.^22^ Similarly, we found that patients with TNBC-LMD were less likely to receive treatment after their LMD diagnosis. Prospective trials specifically targeting TNBC-related LMD are critically needed.

A general observation based on the current data conforms with previous work showing that treatment of any type following diagnosis of BC-LMD improved survival to 6.54 months.^3, 22, 23^ It might be surmised that greater intention to treat would improve survival, as this study found that patients that did not receive any treatment survived a median of 1.07 months. Survival has also been shown to vary when considering diagnostic modality: cytology versus MRI alone.^2^ However, we found no difference in OS based on the diagnostic modality. This contradiction may relate to our exclusion of cases of BC-LMD, which were treated on clinical suspicion of LMD but for which no CSF cytology or MRI evidence of disease was found.^17^ There may also be improving awareness and recognition of typical radiographic LMD features, which increases the sensitivity of MRI in our institution.

To expand systemic treatment affecting CNS and LMD-related cancer, various novel immunotherapy approaches are being assessed in the management of BC-LMD. The use of systemic pembrolizumab^48^ or systemic ipilimumab and nivolumab^49^ showed promise for a variety of LMD patients (most of whom had LMD from BC), but the median survival was only 3.6 months and 2.9 months, respectively. Only 11 patients at our institution received systemic immunotherapy following diagnosis with BC-LMD, and no associated benefit was found. An alternative approach in trials now includes IT bispecific antibody-armed T-cells that can be directed against HER2 (NCT03661424). A similar trial with IT HER2-directed chimeric antigen receptor (CAR) T cells (NCT03696030) is recruiting currently. A phase 3 study is planned to use the systemic administration of blood-CSF penetrant drug ANG1005 (a paclitaxel-peptide conjugate that crosses the blood CSF barrier via a low-density lipoprotein receptor-related protein-1 (LRP-1) mediated transcytosis). A phase 2 study with the same agent showed activity and an average survival of 8 months in BC patients with LMD.^50^ At the time of this report, there are only 23 recruiting or active clinical trials in the US targeting LMD generally and even fewer targeting BC-LMD. Even with improvements in outcomes for HER2 + BC-LMD, the need to find new therapies which improve OS in BC-LMD is dire.

## Data Availability

All data and materials are stored in a safe, protected drive that is available for review.
